# Generalized Necrobiotic Xanthogranuloma in a Patient with Multiple Myeloma

**DOI:** 10.4274/tjh.2013.0364

**Published:** 2014-12-05

**Authors:** Maria Jimenez Esteso, Jose Verdu, Francisco de Paz, Fabian Tarin

**Affiliations:** 1 University General Hospital of Alicante, Clinic of Hematology and Hemotherapy, Alicante, Spain

**Keywords:** Multiple, Myeloma, Skin, Necrobiotic, Xanthogranuloma

## CLINICAL IMAGE IN HEMATOLOGY

**Figure 1:**
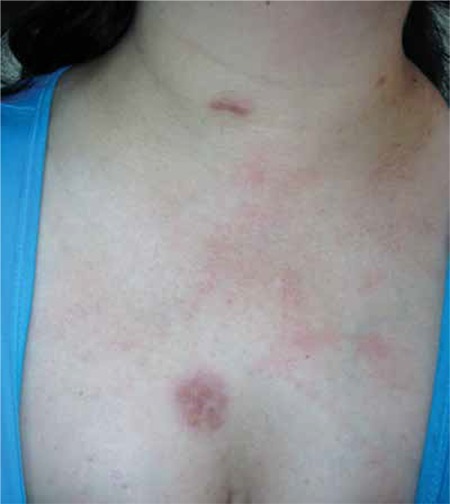
Dermatological lesions.

Analysis of a skin biopsy obtained from a 42-year-old female presenting with multiple yellowish to reddish-brown nodules and plaques on the arm, chest (Figure 1), and abdomen showed that she had generalized necrobiotic xanthogranuloma (Figure 3). Informed consent was obtained.

**Figure 2:**
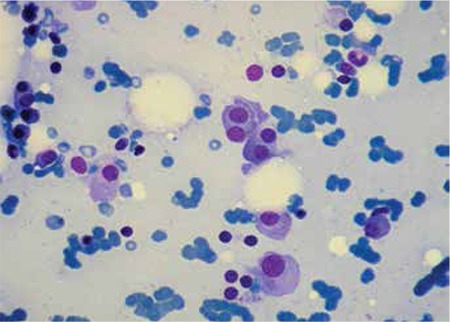
Morphological analysis of the bone marrow aspirate demonstrated abnormal plasma cells.

**Figure 3:**
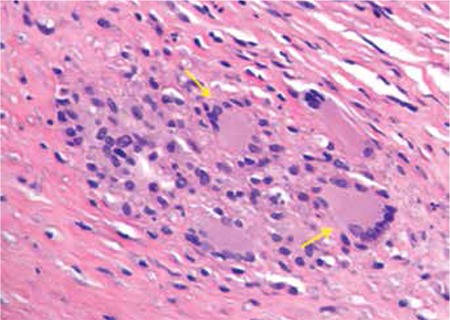
Skin biopsy studies revealed 2 granulomas containing Touton giant cells and foamy histiocytes.

A week later, she was diagnosed with IgG-kappa multiple myeloma (Figure 2).

The laboratory workup showed the following: Hb: 109 g/L, Hct: 0.34 L/L, WBC: 9.5x109/L, Plt: 194x109/L, with normal differential white cell count. Other studied parameters included immunoglobulin IgG:1790 mg/dL (normal range: 750-1750), IgA: 279 mg/dL (normal range: 90-450), IgM: 40 mg/dL (normal range: 70-280); and kappa light chains: 1830 mg/dL (normal range: 629-1320 mg/dL).

The coexistence of paraproteinemias and necrobiotic xanthogranuloma is well described (above all, monoclonal gammopathy of unknown significance), and it would seem reasonable to recommend performing at least serum electrophoresis for patients affected by these rare conditions [1].

**Conflict of Interest Statement**

The authors of this paper have no conflicts of interest, including specific financial interests, relationships, and/or affiliations relevant to the subject matter or materials included.
